# Comprehensive analysis of causal pathogens and determinants influencing black rot disease development in MD2 pineapples

**DOI:** 10.3389/fmicb.2024.1514235

**Published:** 2025-01-21

**Authors:** Manori Kuruppu, Yasmeen Siddiqui, Hala Badr Khalil

**Affiliations:** ^1^Laboratory of Sustainable Agronomy and Crop Protection, Institute of Plantation Studies, University Putra Malaysia, Serdang, Malaysia; ^2^Department of Biological Sciences, College of Science, King Faisal University, Al Hofuf, Saudi Arabia; ^3^Department of Genetics, Faculty of Agriculture, Ain Shams University, Cairo, Egypt

**Keywords:** black rot disease, harvesting index, MD2 pineapple, microenvironment, minimum inoculum concentration, *Thielaviopsis paradoxa*

## Abstract

Malaysia ranks among the world’s top 20 pineapple producers, driven by the success of the MD2 variety in meeting domestic and international demand. However, postharvest losses due to pathological diseases remain a challenge. Black rot, a major postharvest disease, causes significant economic losses in pineapples. Despite its presence in various cultivars, its aetiology, specifically in MD2 pineapples remains unclear. This study was conducted to identify the principal causative pathogen of black rot disease in pineapple from three different regions. In addition, critical factors influencing black rot disease were investigated, such as the minimum inoculum concentration, appropriate storage temperature, and maturity index required to initiate infection. *Thielaviopsis paradoxa* was identified as the primary pathogen causing black rot, with 50 and 45% occurrence at two specific cultivation sites. Other associated pathogens included *Lasiodiplodea theobromae*, *Trichoderma asperellum*, *Curvularia eragrostidis*, *Neoscytalidium dimidiatum*, *Aspergillus assiutensis*, and *Aspergillus aculeatus*. Fruits stored at ambient temperature with a maturity index of 2 showed higher disease progression than those in cold storage. A minimum inoculum concentration of 1 × 10^4^ CFU/mL was sufficient for infection at both storage conditions. The Pearson correlation analysis revealed a weak positive link (*r* > 0.39, *p* < 0.0001) between harvesting index and fruit pH, while pH and storage temperature had a strong positive correlation (*r* = 0.83, *p* < 0.0001). The increments in pH correlated with lesion length and infected area (*r* = 0.83 and *r* = 0.82, respectively). The harvesting index showed a strong positive correlation with the proportion of infected area (*r* = 0.86, *p* < 0.0001). The telomorph state of *T. paradoxa*, identified as *Ceratocystis paradoxa*, persists in soil and decaying plant material, acting as a quiescent pathogen, increasing cross-contamination risks. Urgent measures are required to reduce postharvest losses and maintain the quality of pineapples for international markets.

## 1 Introduction

Pineapple (*Ananas comosus L. Merr*.), a member of the Bromeliaceae family, is among the most economically significant fruit crops cultivated globally (Neri et al., 2021). Historically, it played a pivotal role in the Malaysian economy as the first commodity crop, elevating the country’s global standing in the late 1960s and the early 1970s. Among the cultivated varieties, the MD2 pineapple stands out for its exceptional qualities, including a uniform bright golden color, sweeter taste, higher vitamin C content, lower fiber content, reduced acidity, thinner skin, and superior eating quality, with an average fruit weight of approximately 1.5 kg. These attributes have made the MD2 variety a preferred choice in both the domestic and international markets. However, postharvest losses caused by microbial infections remain a significant challenge, leading to substantial economic losses. Globally, postharvest losses in fruits range from 40 to 50% in developing countries, primarily owing to microbial spoilage (Yao Fulgence et al., 2021). Several postharvest diseases have been documented in pineapples, including black rot, bacterial heart rot, fusariosis, fruitlet core rot, green fruit rot, and leathery pockets (Joy and Sindhu, 2012). The most prominent fruit rot diseases in MD2 are heart rot and black rot, which cause severe economic losses in postharvest losses in pineapple fresh markets and export-oriented industries (Thalip et al., 2015). Black rot is widespread and has been reported in regions across Asia, Africa, the Americas, the Caribbean, Europe, and Oceania, highlighting its global significance.

The disease can manifest within 8–12 h of infection, frequently aided by poor postharvest handling, bruising, and inadequate storage conditions (Majumdar and Mandal, 2018). Black rot symptoms include tan-colored peel, blackened stem-end, and watery pulp with black fungal colonies, sometimes accompanied by a peculiar fruity odor. Infected fruits may also leak through their shell. Warm and rainy weather conditions during harvest and inadequate hygiene in storage facilities contribute to disease development and transmission (Joy and Sindhu, 2012). Despite the significant economic impact of black rot, there is still a major gap in comprehending its epidemiology and aetiology, notably in the MD2 pineapple. Previous research has described symptoms and fundamental pathogen behavior, but specific information about the pathogen’s survival mechanisms, host colonization methods, and the environmental factors which facilitate disease establishment is lacking. Furthermore, while molecular techniques have been used for preliminary pathogen identification, discriminating between similar-looking fungi remains difficult, particularly during the early stages of infection. This knowledge gap impedes the development of focused management methods to reduce post-harvest losses. Furthermore, there is a lack of studies focusing on the associated fungal infections linked with black rot in MD2 pineapples, which may differ from other varieties due to their unique physiological characteristics. The interaction of these pathogens with the MD2 variety’s unique characteristics is still unclear. Addressing this gap is crucial for developing successful postharvest management strategies specific to MD2 pineapples. Therefore, this study intends to address the major gaps in our current understanding of black rot disease in MD2 pineapples through the following objectives: (1) To identify the fungal pathogens that cause black rot in MD2 pineapples, distinguishing them from other stem-end rot pathogens. (2) Investigate the ambient and microclimate conditions that promote pathogen infection, colonization, and proliferation in MD2 pineapples. (3) Investigate the behavior of black rot pathogens during the ripening stage, as well as their survival in plant wastes or field soil. Findings from this study may contribute to the development of sustainable solutions to mitigate postharvest losses, ensuring economic and food security benefits for pineapple growers and industry.

## 2 Materials and methods

### 2.1 Sample collection

Fifty symptomatic MD2 pineapple fruits (index 2, which were 50% yellowish colored peel and focused on the local market), which have a blackish colored stem end portion, blemished tan colored peel with a characteristic fruity odor were selected from storehouses (50 fruits/location) of Ulu Tiram (1^o^36’0” North,103^o^49’0″ N) Johor, Taman Serkam Jaya (2.1476^o^N,102.3851°E), Melaka and Serdang (3.022^o^N,101.7055°E), and Selangor pineapple farms with a cultivation pineapple extent of more than 2acres, using a purposive sampling method for assessing possible fungal pathogens causing black rot disease during January to February 2020. All fruits were harvested 24 h before collection and were ready to be transported by placing them in plastic crates (60 × 40 × 40 cm length × width × height). Each crate had approximately 30 fruits with maturity index 2 and was stored at room temperature (30 ± 2°C) to estimate disease incidence at each location. The fruit samples were placed in an icebox and brought to the laboratory at the Institute of Plantation Studies, University Putra Malaysia, where the study was conducted.

### 2.2 Disease incidence (DI) of black rot diseased fruits in three locations

The disease incidence percentage of three localized fruits was assessed by randomly selected plastic crates, each containing approximately 30 fruits. A total of 30 plastic crates were used for this purpose. This was achieved by counting the total number of symptomatic fruits in each crate.


$$\mathrm{Disease incidence}\;\left(DI\right)=\frac{\mathrm{Number of symptomatic fruits}}{\mathrm{Total number of fruits in each crate}}\times 100$$

### 2.3 Isolation of pathogen

Stem tissue cuttings and decaying leaf cuttings (1 × 1 cm) were obtained from symptomatic MD2 pineapple fruits and necrotic regions of leaves, respectively, and then surface sterilized with 1% sodium hypochlorite aqueous solution followed by three rinses with sterilized distilled water. Sterilized samples were blot-dried under laminar airflow for 15 min. Five pieces of tissue samples were inoculated onto each sterilized PDA plate (Oxoid, United Kingdom) and amended with 0.5 gL^−1^ streptomycin sulphate to prevent bacterial contamination. Simultaneously, the same procedure was followed to obtain fungal colonies from the decaying leaves. For soil pathogen isolation, soil samples were randomly collected from several sites representing pineapple-cultivated areas on Kulim Farm. Ten soil samples (approximately 100 g) from each site (10 cm from the plant base and up to 30 cm in depth surrounding the plant) were collected using a hand fork. Composite samples (100 g) were prepared, placed in an icebox, and transported to the laboratory. Three 5 g soil samples were placed into separate Erlenmeyer flasks, and 50 mL of 0.05% Tween 20 was added and mixed well. The mixtures (50 mL) were kept in a shaker incubator (Protech model SI-50, Malaysia) at 100 rpm for 2 h to homogenize the soil suspension. The serial dilution method was used to dilute the soil suspension to 10^7^ μL. One hundred microlitres of each suspension was spread on PDA (Oxoid) plates with a cell spreader and incubated at 28°C in a shaker incubator for 3 days covered with black polythene to mimic darkness. Isolated pathogen colonies were subjected to two consecutive subcultures. Fully grown colonies were visually observed for morphological features, whereas conidial morphology was observed using a compound microscope (Olympus model BX-50F4; Tokyo, Japan) equipped with Dino-Eye. Seven-day-old pure cultures were used for DNA extraction using the CTAB method. In soil and decaying leaves isolates, each pathogen occurrence percentage was calculated by the following formula (Sampò et al., 1997),


$$\mathrm{Pathogen occurrence}\%=\frac{\mathrm{No}.\mathrm{of relevant confirmed colonies}}{\mathrm{Total}\;\mathrm{no}.\mathrm{of colonies confirmed}}\times 100$$

### 2.4 Fungal DNA isolation

Seven-day-old morphologically distinct single colonies from each categorized group were used for DNA extraction using the cetyltrimethyl ammonium bromide (CTAB) method according to the protocol described by Sambrook et al. (1989), with minor modifications. Briefly, fungal mycelium was harvested and homogenized with glass beads in CTAB extraction buffer (2% CTAB, 100 mM Tris–HCl pH 8.0, 20 mM EDTA, 1.4 M NaCl), followed by incubation at 65°C. The mixture was then treated with chloroform: isoamyl alcohol (v: v, 24:1), and DNA was precipitated using isopropanol. After washing with 70% ethanol, the DNA pellet was resuspended in TE buffer (10 mM Tris–HCl pH 8.0, 1 mM EDTA). The quality and quantity of the extracted DNA were assessed using 1.5% agarose gel electrophoresis, ensuring its suitability for subsequent molecular analyses.

### 2.5 PCR amplification of ITS regions from fungal isolates

The ribosomal internal transcribed spacer (ITS) regions of fungal isolates were used for identification (White et al., 1990; Ho et al., 2016; Borges et al., 2019). Forward (F) and reverse (R) primer sequences were used in the PCR reactions. The ITS gene regions were ITS1F (5’-TCCGTAGGTGAACCTGCGG-3′) and ITS4R (5’-TCCTCCGCTTATTGATATGC-3′). PCR reaction mix (25 μL) was prepared, containing 12.5 μL of 2X Taq PCR Master Mix, 2 μL of each forward and reverse primer, and 9 μL of nuclease-free water. The PCR conditions for ITS amplification were as follows: 94°C for 5 min, followed by 30 cycles of 94°C for 1 min, 52°C for 1 min, and 72°C for 1 min, with a final extension at 72°C for 10 min. PCR was performed using a thermocycler (Biometra, Germany). The PCR products were visualized via electrophoresis on a 2% agarose gel (Sigma Aldrich) and subsequently sent to Apical Scientific Sdn Bhd, Malaysia for sequencing. The resulting sequences were carefully edited to remove low-quality base calls and trimmed to include only high-quality regions.

### 2.6 Fungal isolate identification and GenBank submission

To identify fungal isolates, a comprehensive homology search was conducted using the Basic Local Alignment Search Tool (BLAST) and GenBank database. BLAST results provided the identity of the isolates to the closest fungal species, enabling accurate species identification. Following confirmation of the species, the sequences were prepared for submission to the GenBank database. This process involved formatting the sequences according to GenBank’s submission guidelines. The sequences were then submitted through the BankIt tool provided by NCBI. After a review process by GenBank curators, unique accession numbers were assigned to each submitted sequence, allowing for future reference and retrieval by the scientific community.

### 2.7 Multiple sequence alignment and phylogenetic analysis

DNA sequences of the ITS region from the tested pathogenic fungal isolates were selected to construct a detailed phylogenetic tree. This analysis aimed to enhance understanding of the evolutionary and genetic relatedness of the isolates. For this, DNA sequences were aligned using the CLUSTALW algorithm in MEGA11, with 90% conserved sites (Tamura and Nei, 1993). Initial trees for the heuristic search were generated automatically by applying the neighbor-joining and Bio NJ algorithms to a matrix of pairwise distances calculated using the JTT model. The topology with the highest log-likelihood value was selected. A phylogenetic tree was then constructed using the maximum likelihood method based on the JTT matrix-based model (Tamura et al., 2021), with 1,000 bootstrap replicates for support. A tree was drawn to scale with branch lengths representing the number of substitutions per site. Positions with gaps or missing data were excluded from the analysis. A Bayesian phylogenetic analysis was conducted to also infer the evolutionary relationships among the fungal isolates. Bayesian inference was performed using BEAST2 hosted by CIPRES.^1^ Two independent Markov Chain Monte Carlo (MCMC) runs were performed with trees sampled every 1,000 generations.

### 2.8 Storage conditions and minimum threshold concentration of inoculum density on the progression of black rot disease

This study aimed to investigate the effects of storage conditions on the development of black rot disease in MD2 pineapple. Zero-indexed MD2 asymptomatic fruits (greenish color, flattened eye, physiologically fully matured focusing distance markets) were selected and surface-sterilized with 1% sodium hypochlorite aqueous solution, followed by rinsing with three changes of sterilized distilled water. Fungal inoculum stock was prepared using 5-day-old cultures of *T. paradoxa* as the main causal agent of black rot. Fungal mycelia were scraped by adding sterilized distilled water (10 mL) and filtered. The spore count in the inoculum stock solution was adjusted to 1×10^9^ spores/mL using a hemocytometer. Furthermore, a homogenized stock solution was serially diluted to prepare five differently concentrated aliquots containing spores at 1 × 10^3^, 2.5 × 10^3^, 5 × 10^3^, 1 × 10^4^, and 1 × 10^5^ spores/mL. The experiment was laid out in a completely randomized design (CRD) with two factors: five aliquots with varying concentrations different inoculum concentrations and two different storage conditions with six replicates, each consisting of five healthy MD2 fruits (zero-indexed) as the experimental units. These inoculum concentrations were tested under two separate storage conditions: (1) room temperature conditions (rt) including T1(1 × 10^3^ + rt), T2 (2.5 × 10^3^ + rt), T3 (5 × 10^3^ + rt), T4 (1 × 10^4^ + rt), T5 (1 × 10^5^ + rt) and (2) cold storage (cs) conditions including T6 (1 × 10^3^ + cs), T7 (2.5 × 10^3^ + cs), T8 (5 × 10^3^ + cs), T9 (1 × 10^4^ + cs), and T10 (1 × 10^5^ + cs). Each fresh-cut stem end portion (2.5 cm) was dipped in 10 mL of freshly prepared concentrations of the main causal agent of black rot. The inoculated fruits were incubated under two storage conditions: room temperature (30 ± 2°C and 75% relative humidity [RH]) and cold storage (9 ± 2°C and 85% RH) as mentioned above. Uninoculated fruits served as control. Generally, the shipment of MD2 in Malaysia takes place for approximately 7 days, primarily targeting Asian markets, especially China, and our focus was to maintain the quality of export-oriented MD2 for such a duration; hence, 10 days were selected as the optimum duration to carry out this experiment. Ten days after inoculation, the lesion length of the core was measured using a ruler, and both the total fruit area and infected fruit area were recorded using self-prepared grid lines of 1 cm^2^ from each fruit (30 fruits/treatment). Each fruit in each replicate was used to record the data. The disease severity index percentage (DSI%) and percentage of infected area were recorded based on the severity scoring scale described by Rohrbach and Johnson (2009) and calculated using the formula described by Palou (2018). To confirm pathogenesis, two consecutive experiments were conducted under the same environmental and biological conditions, with six replicates, each consisting of five fruits. The data from the two experiments yielded similar results and were pooled together and treated as a single dataset before analyses.


$$DSI\;\left(\%\right)=\sum \;\left(a+b+c\ldots \right)/\left(N\times Z\right)\times 100$$

Where DSI (%) is the disease severity index, Ʃ (a + b + c…) is the summation score of the diseased fruits, N is the total number of fruits in the sample, and Z is the score of the highest diseased sample.

### 2.9 Harvesting indices on the progression of black rot disease

Two distinct harvesting indices were selected for this experiment, each corresponding to different marketing purposes. The first group, labeled as “zero-index” fruits, were characterized by their fully green color and flattened eye shells, indicating physiological maturity despite their green appearance. The second group, referred to as “index 2” fruits, were 50% yellow-shelled and suitable for fresh consumption, representing a ripeness level ideal for eating. The fruits were surface-sterilized by immersing them in a 1% sodium hypochlorite solution for 5 minutes, followed by rinsing with three changes of sterilized distilled water. The surface-sterilized fruits, from the two selected categories, were then inoculated by dipping the stem-end portion (2.5 cm in length) into a spore suspension containing 1 × 10^4^ spores/mL of the primary black rot pathogen for 10 min. All inoculated fruits were subsequently incubated at room temperature for 10 days. The disease severity index percentage (DSI%) and percentage of infected area were calculated according to Palou (2018). Two consecutive experiments were conducted using a completely randomized design (CRD) with two treatments, representing two different harvesting indices. Each treatment was replicated six times, with each replicate consisting of five fruits, resulting in data collection from 30 fruits per treatment.

### 2.10 Relationship of host factors and micro-environmental factors on black rot disease development in pineapple fruit

The disease triangle highlights that disease development primarily depends on host characteristics, including factors like the fruit harvesting index (maturity level), fruit pH, and external environmental conditions such as inoculum density and storage temperature. Pearson correlation analysis was employed to assess the relationships between these factors and their influence on the development of black rot disease symptoms, providing insight into their behavioral patterns in disease progression.

### 2.11 Statistical analyses

Two-way ANOVA was performed for data analysis in a completely randomized design (CRD) carried out with two factors, namely, two storage conditions and five inoculum densities. One-way ANOVA was performed for data analysis in CRD to assess disease progression in the two harvesting stages. Collected data were compiled in two consecutive experiments and checked for homogeneity using the Shapiro test before statistical analysis using SAS software (version 9.4). Mean separation was performed using the least significant difference (LSD) test at a confidence level of 95%. Correlation analysis was performed for the available data regarding host and micro-environmental factors affecting disease progression.

## 3 Results

The incidence of black rot on MD2 pineapple fruits collected from three locations in Malaysia was analyzed to determine disease prevalence in these regions. The results indicated a significant (*p* ≤ 0.05) variation in disease prevalence across the regions. The analysis revealed that the highest disease incidence percentage (DI%) was observed in fruits collected from Johor (89.1%), followed by those collected from Melaka (57.3%) and Selangor (29.3%).

**Figure 1:**
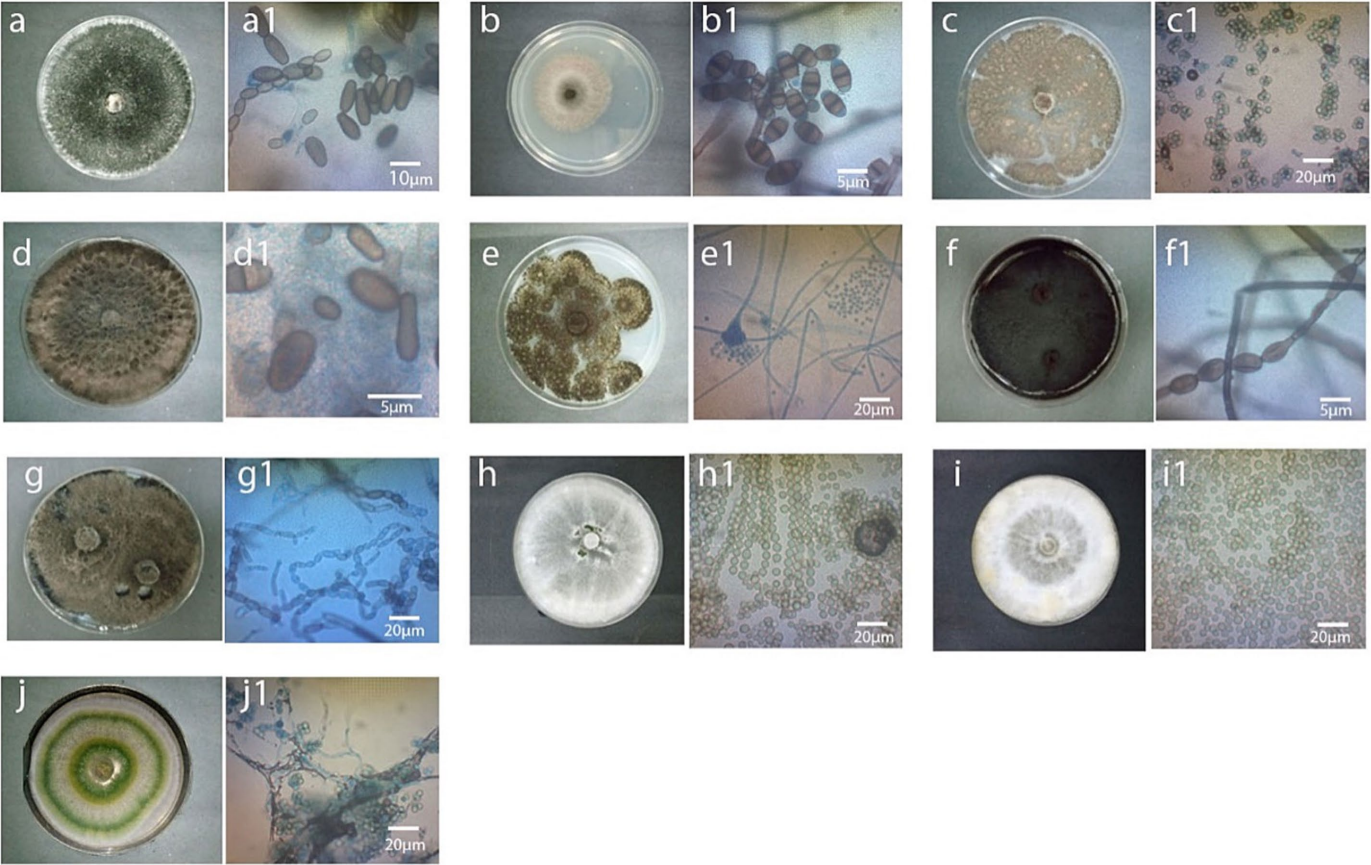
Colony and spores of fungi associated with diseased MD2 pineapple fruits 10 days after storage at room temperature. From top left to right, **(A)** *T. paradoxa* (JA4/MP2), a1; spore arrangement of JA4/MP2, **(B)** *C. eragrostidis* (JA3/MA3), b1; spore arrangement of JA3/MA3, **(C)** *A. assiutensis* (MA1/SA3), c1; spore arrangement of MA1/SA3, **(D)** *L. theobromae* (MP1), d1; spore arrangement of MP1, **(E)** *A. aculeatus* (JA1), e1; spore arrangement of JA1, **(F)** *N. dimidiatum* (SP2), f1; spore arrangement of SP2, **(G)** *N. hyalinum* (SP1), spore arrangement of SP1, **(H)** *T. erinaceum* (MA2), spore arrangement of MA2, **(I)** *T. strigosellum* (SA4), spore arrangement of SA4, **(J)** *T. asperellum* (JA2), j1; spore arrangement of JA2 (x400).

**Figure 2:**
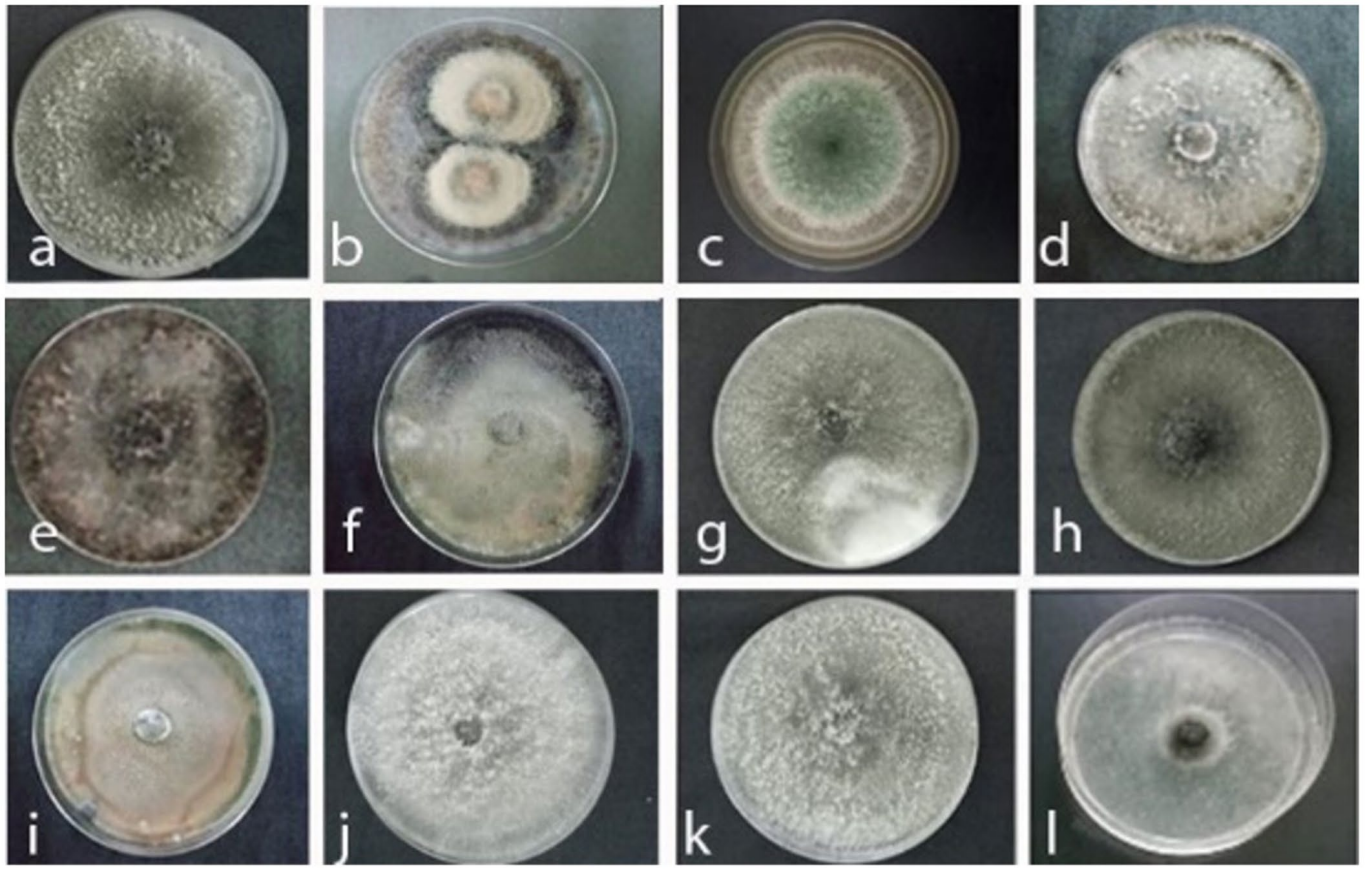
Colony appearance of black rot pathogens and associated fungi isolated from diseased MD2 pineapple fruits, decaying leaves, and soil samples collected from pineapple fields in Johor top left to right. **(a)** *Thielaviopsis paradoxa* JA4, **(b)** *Curvularia eragrostidis* JA3, **(c)** *Trichoderma asperellum* JP2, **(d)** *Lasiodiplodea theobromae* JDL1, **(e)** *Lasiodiplodea theobromae* JDL3, **(f)** *Thielaviopsis ethacetica* JDL5, **(g)** *Thielaviopsis paradoxa* JDL 7, **(h)** *Ceratocystis paradoxa* JDL2, **(i)** *Fusarium* spp. J9, **(j)** *Ceratocystis paradoxa* J2, **(k)** *Thielaviopsis paradoxa* J11, (l) *Thielaviopsis paradoxa* J12.

**Figure 3:**
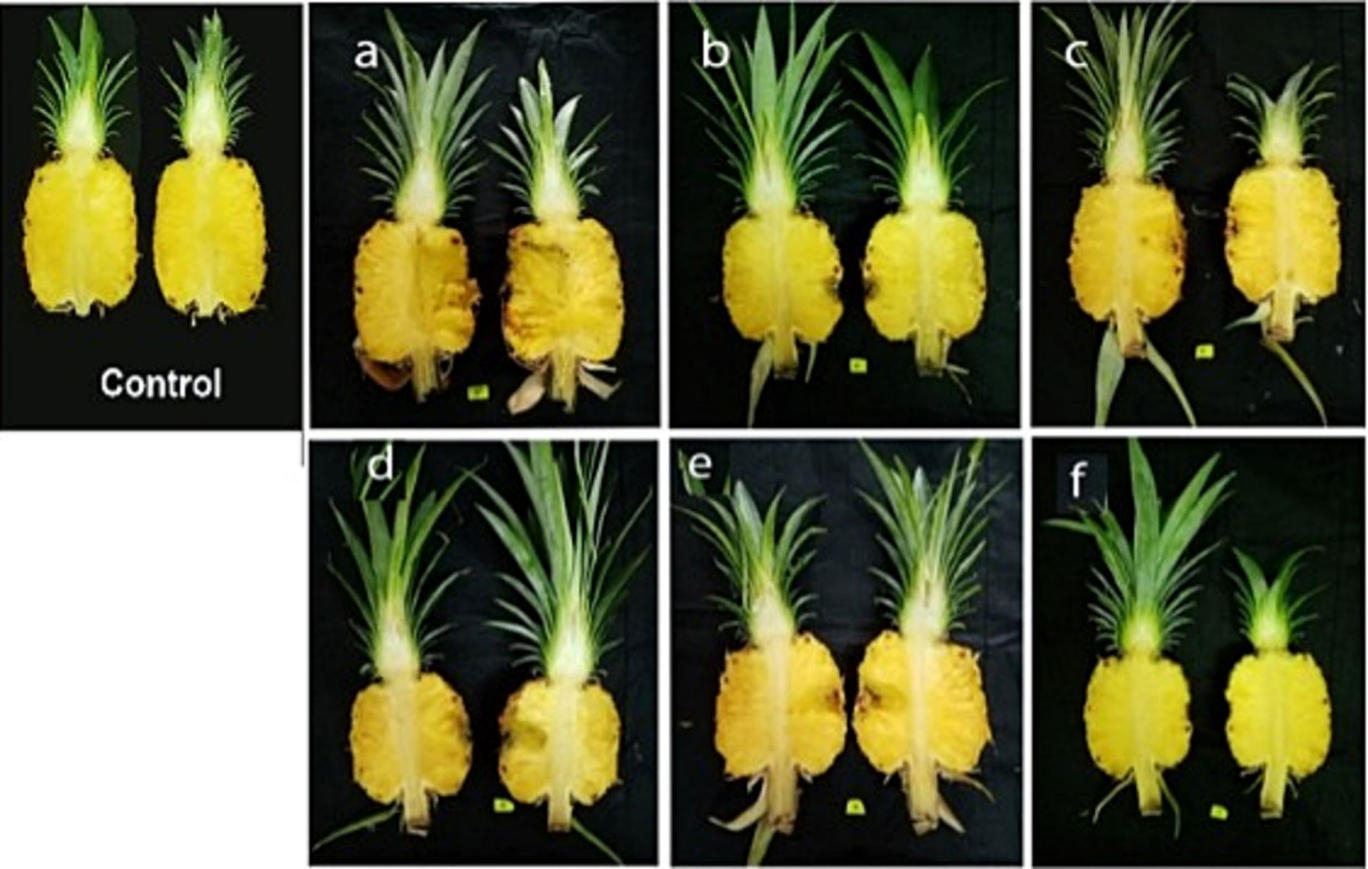
Pathogenesis of *T. paradoxa* validated with associated fungi of black rot diseased MD2 fruits 10 days after storage at room temperature. The order of the tested fungi was as follows: **(A)** *Thielaviopsis paradoxa*, **(B)** *Aspergillus assiutensis*, **(C)** *Curvularia eragrostidis,* **(D)** *Neoscytalidium dimidiatum*, **(E)** *Lasiodiplodea theobromae,* and **(F)** *Aspergillus aculeatus*.

The selected diseased pineapple fruits exhibited typical black rot symptoms, including blackish stem ends, blemished brown skin, and softened flesh with a characteristic fruity odor. Forty-one colonies were obtained; 20 from Johor, 11 from Melaka, and 10 from Selangor, each exhibiting distinct visual colony appearances was subjected to molecular identification. For Johor isolates, 10 colonies initially exhibited whitish-gray mycelium with radial growth, which later developed into black velvety colonies. These were confirmed as *Thielaviopsis paradoxa* (JA4, GenBank accession number: MW082788), identified as the most prominent pathogen, with a 50% occurrence rate (Table 1A and Figures 1A, 2A). The remaining 10 colonies were identified as associated fungi of black rot diseased fruits and were classified as follows: (1) *Aspergillus aculeatus* (JA1, GB: MW082785), five colonies with 25% occurrence (Table 1 and Figure 1B); (2) *Curvularia ergrostidis* (JA3, GB: MW082787) three colonies with 15% occurrence Table 1A and Figures 1C, 2B and (3) *Trichoderma asperellum* (JA2, GB: MW082791) two colonies with 10% occurrence Table 1A and Figures 1D, 2C. Of the 11 colonies isolated from the Melaka samples, five colonies with 45.4% occurrence were identified as *T. paradoxa* (MP2, MW082790), and the remaining colonies were identified as *Aspergillus assiutensis,* 1 colony with 9% occurrence (MA1, GB: MW082792), *Trichoderma erinaceum,* 1 colony with 9% occurrence (MA2, GB: MW082793), *Curvularia eragrostidis* 2 colonies with 18% occurrence (MA3, GB: MW082794), and *Lasiodiplodea theobromae* 2 colonies with 18% occurrence (MP1, GB: MW082796). Whereas for Selangor, out of 10 colonies, 50% were identified as *Neoscytalidium dimidiatum* (SP2, GB: MW082810), 30% as *Neoscytalidium hyalinum* (SP1, GB: MW082809), and 10% as *Aspergillus assiutensis* (SA3, GB: MW082805) and 10% as *Trichoderma strigosellum* (SA4, GB: MW082808) (Table 1 and Figure 1).

**Table 1:** Molecular identification and colony and spore morphology of fungi isolated from diseased MD2 pineapple fruits collected from farmers’ fields across three locations.

Isolate	Fungi	Location of diseased fruit samples collected	Colony morphological characters	Spore characters	Accession numbers
JA4	*Thielaviopsis paradoxa*	Johor	Whitish-gray colony with radial growth and later turned into a black velvety appearance	Thick-walled resting spores (chlamydospores) and two types of conidia such as micro conidia (hyaline, cylindrical and small-sized, arranged in a chain) and macro conidia (6.5 × 2.5 μm) which big, brown colored and oval shaped	MW082788
JA1	*Aspergillus aculeatus*	Johor	Gray to brownish color circular several colonies and protuberant in canters	Consists with phialides and bears ellipsoidal conidia borne in radiate heads	MW082785
JA2	*Trichoderma asperellum*	Johor	Greenish color arranged as concentric halos and compact tuft colony	Conidiophores are repeatedly branched and arranged in whorls	MW082791
JA3	*Curvularia eragrostidis*	Johor	Cottony gray color mycelia with concentric rings on the plate	Conidia are straight and fusiform in shape with three septa and the middle septa is darker than others	MW082787
MA1	*Aspergillus assiutensis*	Melaka	Blackish color, suede-like surface	Colony surface consists with densely compacted conidiophores	MW082792
MA2	*Trichoderma erinaceum*	Melaka	Cottony whitish color appearance and then turns into a slight greenish color	Single celled conidia with large numbers	MW082793
MA3	*Curvularia eragrostidis*	Melaka	Cottony gray color mycelia with concentric rings on the plate	Conidia are straight and fusiform in shape with three septa and the middle septa is darker than others	MW082794
MP1	*Lasiodiplodea theobromae*	Melaka	Initially whitish color mycelium then turns into blackish color fluffy colony	Oval-shaped spores that present a thick cross wall in the middle	MW082796
MP2	*Thielaviopsis paradoxa*	Melaka	Whitish-gray colony with radial growth and later turned into a black velvety appearance	Thick-walled resting spores (chlamydospores) and two types of conidia such as micro conidia (hyaline, cylindrical and small-sized, arranged in a chain) and macro conidia (6.5 × 2.5 μm) which big, brown colored, and oval shaped	MW082790
SA3	*Aspergillus assiutensis*	Selangor	Blackish color, suede-like surface	Colony surface consists with densely compacted conidiophores	MW082805
SA4	*Trichoderma strigosellum*	Selangor	Whitish color tuft colony	Single celled conidia with large numbers	MW082808
SP1	*Neoscytalidium hyalinum*	Selangor	White hyaline colonies in early stages and then turn into wooly cottony olive green to grayish color colonies	Anthroconidia were ellipsoid to ovoid or round shaped and arranged as a chain	MW082809
SP2	*Neoscytalidium dimidiatum*	Selangor	White hyaline colonies in early stages and then turn into wooly cottony olive green to grayish color colonies	Branched mycelia have zero to one septate anthrospores and anthroconidia were ellipsoid to ovoid or round shaped and arranged as a chain	MW082810

Johor was identified as the region with the highest incidence of the disease, prompting the selection of this state for further investigation. Consequently, this study was extended to focus on samples collected from Johor to understand the underlying factors and patterns contributing to this elevated disease incidence. The isolates collected from decaying pineapple leaves and soil cultivated at Johor Kulim farm yielded 25 and 10 colonies, respectively. Out of 25 colonies isolated from decaying leaves, three colonies were identified as *L. theobromae* (JDL1, GB: MZ496555), with similar colony morphology, representing 12% of the total colonies Table 2 and Figure 2D. Additionally, two colonies with different colony appearance were also identified as *L. theobromae* (JDL3, GB: MZ496627) with 8% occurrence (Table 2 and Figure 2E). The remaining 20 colonies were categorized as; 10 colonies each of *T. paradoxa* (JDL5, GB: MZ496629) (Table 2 and Figure 2F) and *T. ethacetica* (JDL7, GB: MZ 496633) (Table 2 and Figure 2G), together representing occurrence of 80%. In contrast, for soil samples, 10 pure colonies with distinct morphological features were isolated. Two colonies were morphologically identified as *T. paradoxa* (JDL2, GB: MZ496626) (Table 2 and Figure 2H), with an occurrence of 20%. Two colonies were identified as *Fusarium* spp. (J9, GB: MZ505640) (Table 2 and Figure 2I), with a similar occurrence to former colonies. The remaining six colonies were identified as *Ceratocystis paradoxa* (J2) (Table 2 and Figure 2J), two colonies as *T. paradoxa* (J11) (Table 2 and Figure 2K), and two colonies as *T. paradoxa* (J12) (Table 2 and Figure 2L) each representing 20% of the total. However, they did not achieve the required purity level for deposition into GenBank to assign their accession numbers.

**Table 2:** Molecular identification and the colony morphology of the fungal isolates isolated from decaying leaves and soil samples collected from pineapple cultivation in Kulim Farm, Johor.

Isolate	Pathogen	Colony morphology	Gene bank accession number	Occurrence (%)
Decaying leaves				
JDL1	*Lasiodiplodea theobromae*	early whitish gray color colony turn into fully gray within 5–7 days and fluffy appearance	MZ496555	12
JDL3	*Lasiodiplodea theobromae*	early whitish gray color colony turn into fully gray within 3–5 days and fluffy appearance	MZ496627	8
JDL5	*Thielaviopsis paradoxa*	whitish gray radial growth colony turned into blackish color within 3–5 days with a velvety appearance	MZ496629	80
JDL7	*Thielaviopsis ethacetica*	Whitish gray radial growth colony turned into partially blackish color within 5–7 days with a velvety appearance	MZ496633	80
Soil				
JDL2	*Thielaviopsis paradoxa*	whitish color colony partially turn in to blackish color within 3–5 days	MZ496626	20
J2	*Ceratocystis paradoxa*	whitish color colony partially turn in to blackish color within 3–5 days	–	20
J9	*Fusarium* spp.	whitish color scattered appearance colony	MZ505640	20
J11	*Thielaviopsis paradoxa*	Whitish color colony partially turn in to blackish color within 3–5 days	–	20
J12	*Thielaviopsis paradoxa*	Whitish color colony partially turn in to blackish color within 3–5 days	–	20

The pathogenesis of black rot disease was comprehensively established through an experiment involving inoculation of the isolated fungi onto healthy asymptomatic fruits that were at the same maturity level (Index 2). This pathogenicity test allowed for the precise identification of the primary causal agent and validation of the pathogen responsible for the disease. Inoculating pineapple fruits with various fungal isolates revealed that *T. paradoxa* is the primary causal agent of black rot disease. The severity of the disease caused by *T. paradoxa* was visually assessed according to the scoring scale and the symptoms were found to be significantly higher in fruits which were artificially inoculated with *T. paradoxa* than those caused by the other tested fungi (Figure 3). The pathogen was re-isolated from the artificially inoculated fruits and identified as *T. paradoxa* fulfilling Koch’s postulates. To further validate these findings, the pathogenesis was also confirmed with associated fungi, including *A. assiutensis, C. eragrostidis, N. dimidiatum, L. theobromae*, and *A. aculeatus* that were formerly isolated from black rot diseased MD2. This validation underscored the primary role of *T. paradoxa* in the development and progression of black rot.

**Figure 4:**
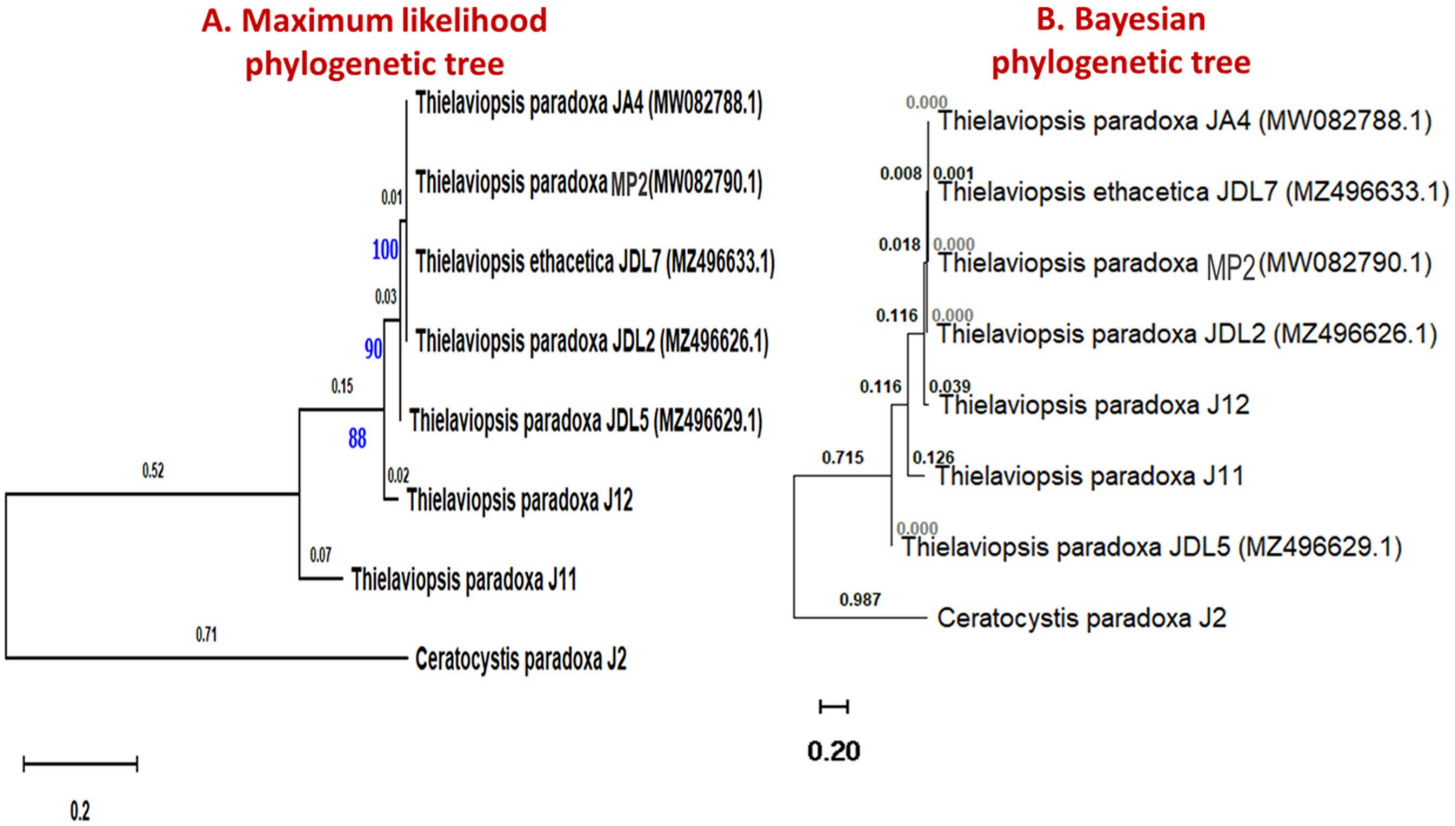
Phylogenetic trees representing the evolutionary relationship of *Thielaviopsis* spp. and *Ceratocystis* spp. isolated from diseased pineapple fruits, decaying leaves, and soil samples from pineapple fields. **(A)** The evolutionary relationship was inferred using the maximum- likelihood method. The tree was built using concatenated sequences of ITS with 1,000 bootstrap replicates. **(B)** Phylogenetic tree of ITS sequences of fungi generated using Bayesian inference. The tree was constructed using BEAST2.

To understand the evolutionary relationships among black rot disease fungal pathogens, a phylogenetic tree was constructed using ITS region sequences from *Thielaviopsis* and *Ceratocystis* fungal isolates (Figure 4). This analysis can provide insights into their genetic diversity, potential virulence factors, and adaptability to different environments. The analyses revealed distinct clades that separated the isolates from the *Ceratocystis* outgroup. Notably, the *Thielaviopsis* isolates formed two major clades. The first clade included *T. paradoxa* isolates JA4, JP1, JDL2, and JDL5, as well as *T. ethacetica* JDL7. All exhibited high bootstrap support values, particularly at nodes leading to JA4 (100%), JDL7 (90%), and JDL2 (88%), indicating strong genetic similarity within this group. The second clade consisted of isolates *T. paradoxa* J11 and J12, which showed a more divergent relationship compared to the first. The outgroup *C. paradoxa* J2 was the most distantly related to the *Thielaviopsis* isolates, establishing its role as a phylogenetically distinct group. The Bayesian phylogenetic analysis validated the results obtained from the maximum likelihood method, providing strong support for the clades of fungal isolates. The clustering of these isolates highlighted potential evolutionary relationships that could be linked to pathogenicity or geographic distribution.

Five different inoculum concentrations and two storage conditions were tested for the infection and progression of black rot disease. The results showed a significant interaction effect between inoculum concentration (I) and storage conditions (E) on mean lesion length (cm) and % of the mean infected area, but no significant effect on DSI (%) (Table 3). The main factors that were elaborated as different inoculum concentrations and two storage conditions were highly significant in the mean lesion length and percentage of the mean infected area. The lesion length, percentage of infected area, and DSI (%) of inoculated fruits were higher in fruits incubated at room temperature than in those incubated under cold storage conditions. The highest lesion length, percentage of infected area, and DSI (%) were observed in T5 (1 × 10^5^ spore/mL), which was exposed to the maximum number of colony-forming units of the pathogen. T2 (2.5 × 10^3^ spore/mL) and T3 (5 × 10^3^ spore/mL) showed equal contributions to lesion length, percentage of infected area, and DSI (%). There were no visual symptoms on the fruits in T1, which consisted of the least concentrated inoculum (1 × 10^3^ spore/mL). The fruits treated with T4 (1 × 10^4^ spore/mL) showed moderate lesion length (%) of the infected area and DSI (%).

**Table 3:** Main and interaction effects of two storage conditions and five inoculum densities on lesion length, percentage of infected area, and DSI (%) of pathogen-inoculated MD2 pineapple (index 0) fruits 10 days after inoculation.

Factors	Lesion length (cm)	% of the infected area	DSI%
Storage conditions (E)			
Room T^o^ (30 ± 2°C) +75% RH	3.9^a^ ± 3.54**	19.07^a^ ± 21.66**	43.71^a^ ± 34.26*
Cold storage T^o^ (9 ± 2°C) +85% RH	0.28^b^ ± 0.66	0.47^b^ ± 1.17	4.35^b^ ± 8.56
LSD at α0.05	0.54	2.76	14.1
Inoculum density (I) (spore/mL)			
T1-1×10^3^	0^d^	0^c^	0^c^
T2-2.5×10^3^	1.18^c^ ± 2.20	3.36^c^ ± 6.7	13.8^cb^ ± 17.00
T3-5×10^3^	1.51^c^ ± 1.99	2.65^c^ ± 4.4	18.5^cb^ ± 19.43
T4-1×10^4^	2.55^b^ ± 2.55	13.6^b^ ± 15.1	26.82^b^ ± 33.68
T5-1×10^5^	5.22^a^ ± 4.22**	29.25^a^ ± 27.26**	67.53^a^ ± 38.68*
CV%	6.74	6.17	4.81
LSD at α0.05	0.85	4.36	22.2
Interaction (E × I)	**	**	ns

Means with the same letters within the column are not significantly different at α0.05. **Highly significant; *Significant.

**Figure 5:**
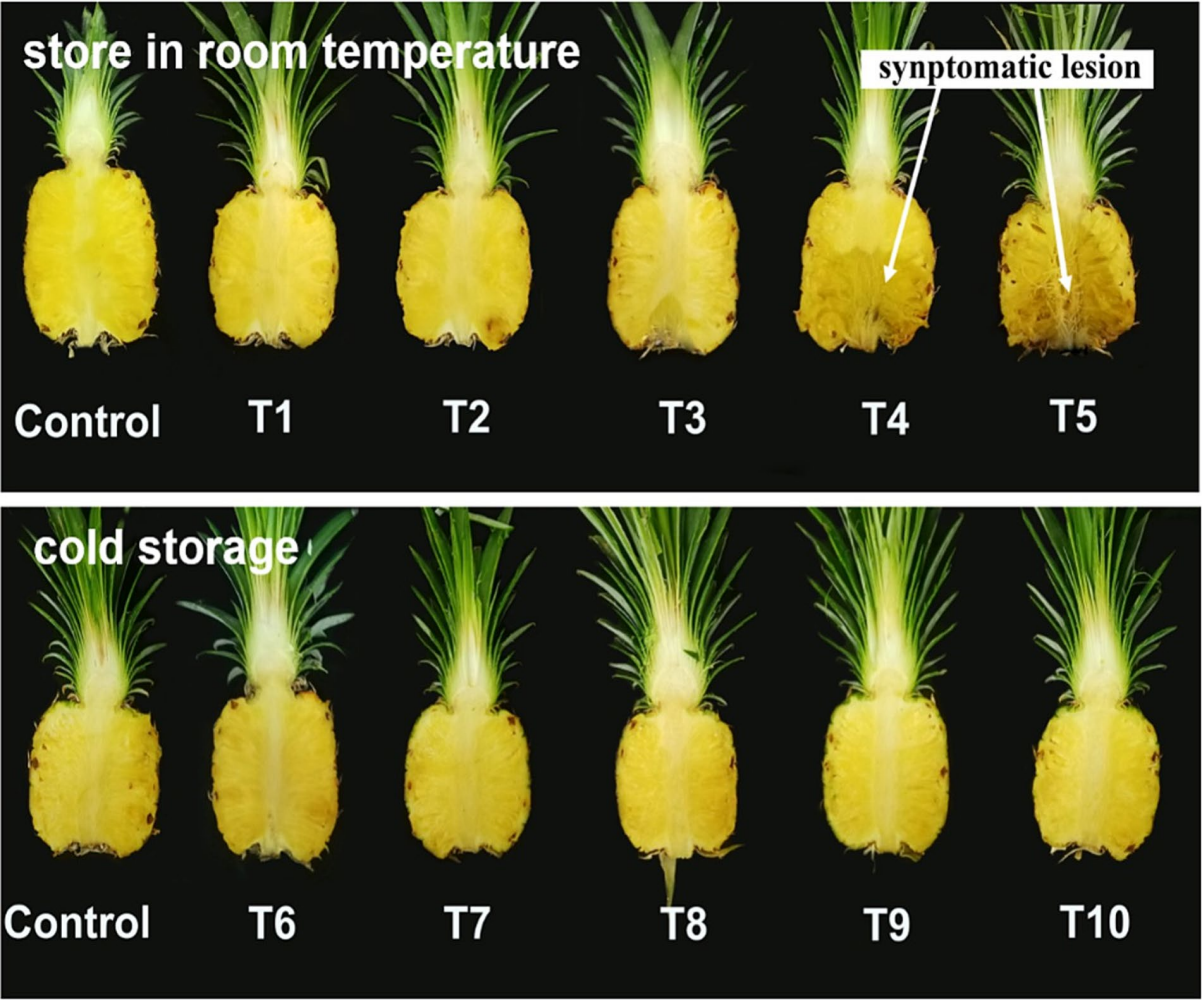
Index 0 MD2 pineapple fruits inoculated with five different inoculum concentrations and stored at two different temperatures for 10 days.

The mean lesion length and percentage of the mean infected area that showed a significant interaction effect were analyzed for the treatment combinations to select the optimal combination for disease progression in the inoculated fruits (Table 4). The treatment combinations demonstrated a highly significant effect on mean lesion length and percentage of mean infected area in inoculated fruits (Figure 5). Among all the parameters, T5 consisted of the maximum colony-forming units and stored under room temperature conditions showed a significant difference from the other treatments exhibiting high disease symptoms. There was no significant difference in mean lesion length and percentage of mean infected area under T2 and T3, which were exposed to different inoculum densities, such as 2.5×10^3^ and 5×10^3^ spore/mL but stored under room temperature conditions. There were no visible symptoms in the observed parameters under T6, T7, and T8, which were exposed to different inoculum densities such as 1 × 10^3^, 2.5 × 10^3^, and 5 × 10^3^ spore/mL, but stored under cold storage conditions. T1 and T6, where fruits were treated with minimum inoculum concentration (1 × 10^3^ spore/mL), showed no symptom development despite being stored under different conditions, such as room temperature and cold storage, respectively. There was a significant difference in the parameters between T2 and T7, and between T3 and T8, when they were treated with an equal concentration of inoculum but stored under different conditions (room temperature and cold storage conditions). A moderate effect was observed in fruits under T4 (1 × 10^4^ spore/mL), but the effect was significant in parameters under T9, where equal inoculum concentrations were used but stored at room temperature and cold storage conditions, respectively. T5 and T10 showed a significant difference in mean lesion length and the % of the mean infected area, and the fruits under these treatments were exposed to the same inoculum densities (1 × 10^5^ spore/mL) but stored in different micro-environmental conditions. An inoculum density of 1 × 10^4^ spore/mL was considered the minimum threshold concentration that contributed to the development of disease symptoms in the fruits under both micro-environmental conditions. This concentration was used to inoculate the fruits in subsequent experiments.

**Table 4:** Five different inoculum concentrations and two different storage conditions as a combination effect on the mean lesion length of the core and percentage of the mean infected area of inoculated MD2 (index 0) fruits.

Factor combination	Mean lesion length of the core (cm)	% of the mean infected area
T1	0^e^	0^e^
T 2	2.3^c^ ± 2.867	6.46^c^ ± 9.018
T 3	2.75^c^ ± 2.191	4.67^dc^ ± 5.442
T 4	4.93^b^ ± 1.295	23.9^b^ ± 16.422
T 5	9.2^a^ ± 0.788**	53.76^a^ ± 7.04**
T 6	0^e^	0^e^
T 7	0^e^	0^e^
T 8	0^e^	0^e^
T 9	0.56^ed^ ± 0.698	0.86^de^ ± 0.9938
T 10	1.05^d^ ± 0.956	1.97^de^ ± 1.646
CV% LSD atα 0.05	6.88 0.83	6.08 4.15

Means with the same letters within the column are not significantly different at α 0.05. **highly significant. T 1-1×10^3^ spore/mL + rt, T 2–2.5×10^3^ spore/mL + rt, T 3-5×10^3^ spore/mL + rt, T 4-1×10^4^ spore/mL + rt, T 5-1×10^5^ spore/mL + rt, T 6-1×10^3^ spore/mL + cs, T 7–2.5×10^3^ spore/mL + cs, T 8-5×10^3^ spore/mL + cs, T 9-1×10^4^ spore/mL + cs, T 10-1×10^5^ spore/mL + cs.

**Figure 6:**
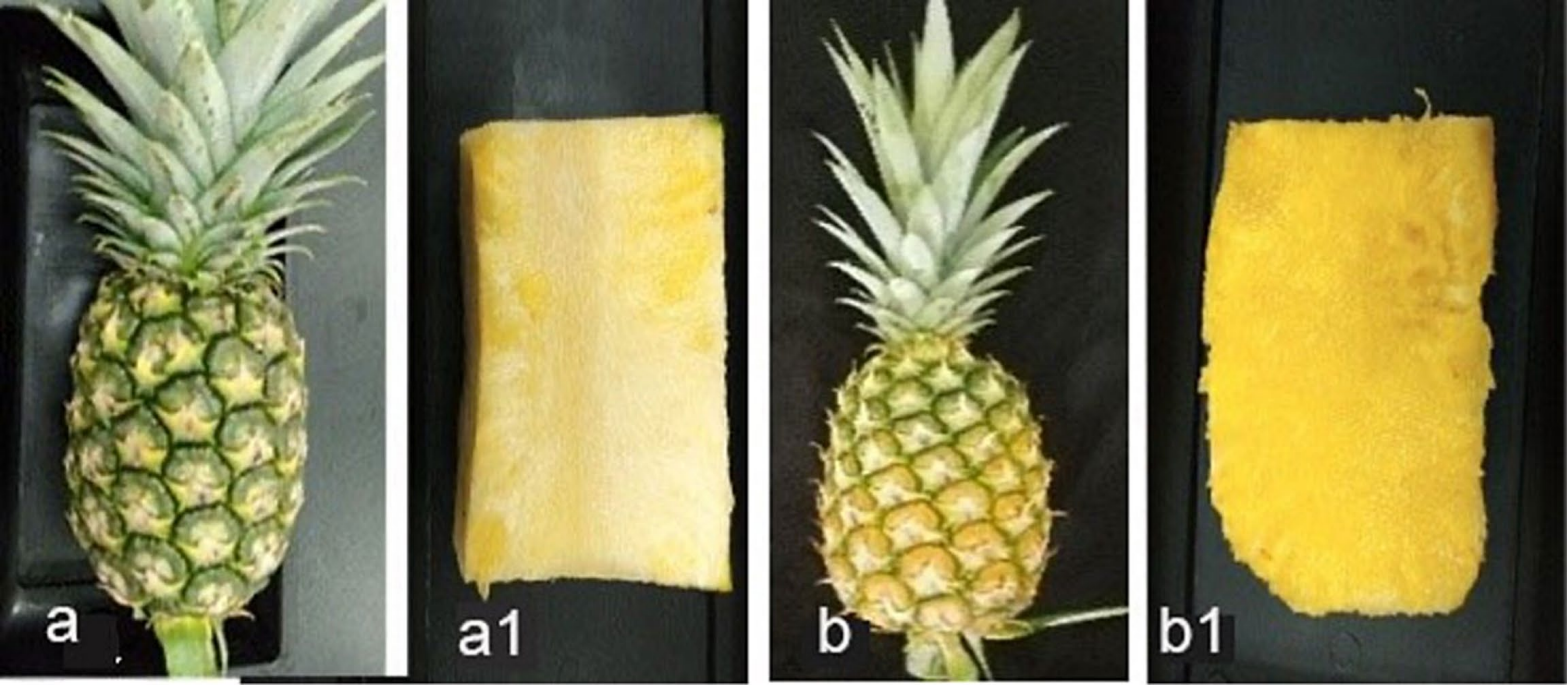
**(A)** Index 0 fruits with greenish color shell and flattened eye (physiologically matured), **(A1)** longitudinal section of index 0 fruit, **(B)** index 2 fruits with 50% yellowed shell and flattened eye, **(B1)** longitudinal section of index 2 fruit.

**Figure 7:**
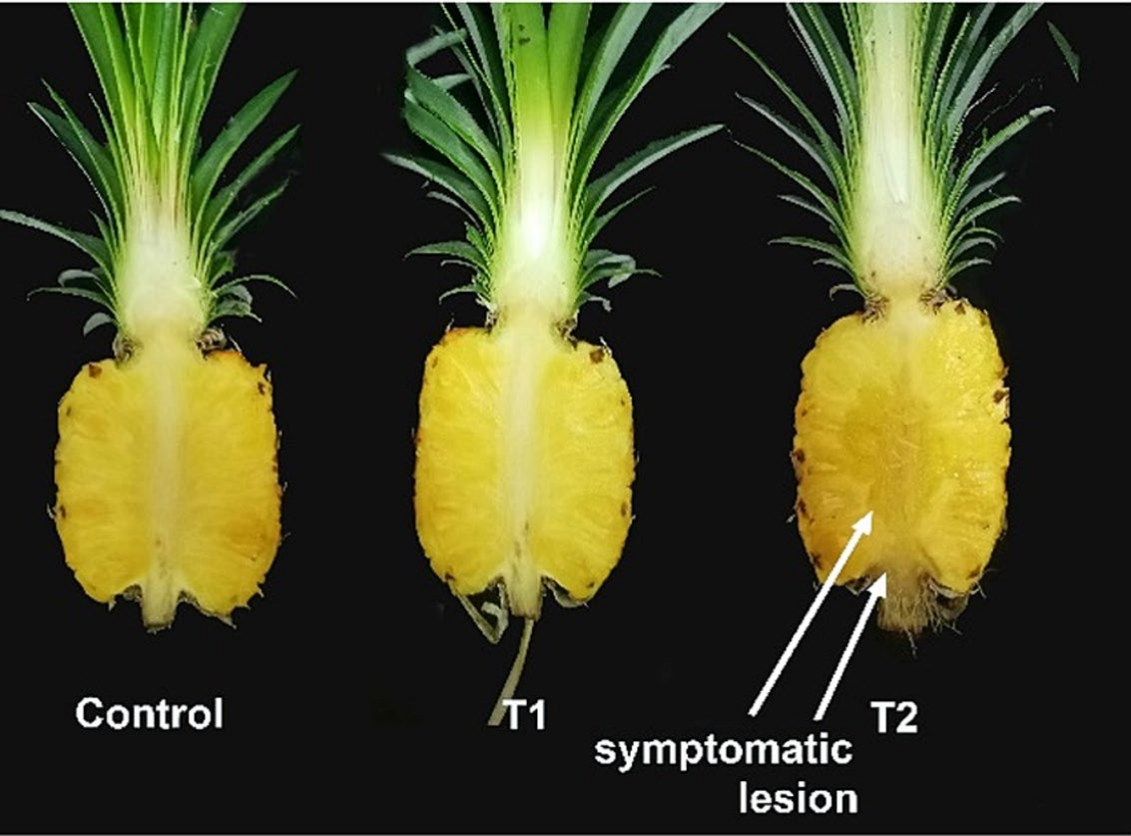
MD2 pineapple fruits at two different harvesting indices as index 0 (T1) and index 2 (T2), inoculated with 1×10^4^ spore/mL of *T. paradoxa* and stored at room temperature conditions for 10 days.

Two harvesting indices: index 0 (T1) and index 2 (T2) fruits (Figure 6), focusing on export fruit markets and local fresh fruit markets, respectively were selected for this experiment. There was a highly significant difference in DSI (%), mean lesion length, and the percentage of mean infected area of inoculated fruits between T1 and T2 (Table 5). The initial pH values of the two fruit indices before inoculation showed a significant difference at a 95% confidence level. The highest mean lesion length, the percentage of the infected area and DSI (%) were observed under index 2 fruits (T2) (Figure 7) while the mean pH of index 2 fruits was higher than index 0 fruits (Table 6).

**Table 5:** The effect of two different harvesting indices of MD2 fruits on mean lesion length of the core, percentage of mean infected area and DSI (%) after inoculating with pathogen and stored at room temperature conditions and pH changes with harvested fruits in selected maturity indices.

Treatments	Mean lesion length (cm)	% of the mean infected area (cm^2^)	DSI%	Mean pH
T1	1.63^b^ ± 0.212	3.57^b^ ± 0.748	33.3^b^ ± 0	4.24 ± 0.159^b^
T2 CV%	9.1^a^ ± 1.744** 4.46	24.9^a^ ± 8.963** 2.35	75.5^a^ ± 8.357* 10.8	4.36 ± 0.123^a^* 3.31
LSD%	0.92	4.7	13.3	0.106

The means followed same letters within a column are not significantly different at α0.05. **highly significant, *significant T1, *T. paradoxa* inoculated fruits in index 0; T2, *T. paradoxa* inoculated fruits in index 2.

**Table 6:** Pearson correlation to depict the relationship between host and external factors on progressing the black rot disease.

	T^o^	ID	pH	HI	IA	LL
T^o^	1					
ID	0.1	1				
pH	0.83***	0.1	1			
HI	0.2	0.1	0.36	1		
IA	0.59**	0.52**	0.83***	0.86***	1	
LL	0.57**	0.54**	0.82***	0.86***	0.92***	1

T, storage temperature; ID, inoculum density; pH, acidity of the pulp; HI, harvesting index (maturity level); IA, the percentage of infected area; LL, lesion length of the core.

The Pearson product correlation between the harvesting index and fruit pH showed a weak positive correlation (*r* ˃ 0.39, *p* < 0.0001). Hence, it means that with an increasing harvesting index (maturity level) of fruit, there is a slight increment of fruit pH, although it is not statistically significant. However, there was a strong positive and statistically significant relationship between the pH and storage temperature (*r* = 0.83, *p* < 0.0001). Simultaneously, both the percentage of the infected area and the lesion length of the core have been reported with increments of pH with the values *r* = 0.83 and 0.82 at *p* < 0.0001, respectively. Further, the results indicated that a strong positive correlation is found between the harvesting index and the percentage of infected area (*r* = 0.86, *p* < 0.0001). A similar relationship was observed between the harvesting index and lesion length of the core of infected fruits. Meanwhile, the Pearson product correlation of storage temperature and the percentage of infected area was found to be moderately positive and significant (*r* = 0.59, *p* < 0.0001). In addition, a moderately positive and significant correlation (*r* = 0.57, *p* < 0.0001) between storage temperature and lesion length of the core of fruit was observed. Thus, it indicated that an increment in storage temperature would lead to a higher disease symptom in inoculated fruits. There was a moderately positive and significant correlation (*r* = 0.54, *p* < 0.0001) between inoculum density and lesion length of the core of fruit as well as between inoculum density and the percentage of infected area (*r* = 0.52, *p* < 0.0001). The results indicated that visualized disease symptoms representing the percentage of the infected area and the lesion length of the core would increase with increasing inoculum density of the pathogen. Moreover, there was a strong positive and statistically significant correlation between the percentage of the infected area and the lesion length of the core (*r* ˃ 0.92, *p* < 0.0001), which represented the progression of black rot disease symptoms in inoculated pineapple fruits (Table 6).

## 4 Discussion

The highlighted results concisely elaborate on the requirements for better establishment and colonization of *T. paradoxa* which is the most predominant pathogen exhibiting high pathogenesis in harvested MD2 pineapple fruits, focusing on both export and local fresh fruit markets in Malaysia. The colony and spore morphology of *T. paradoxa* isolated from black rot-infected MD2 fruits are morphologically similar to the same pathogen isolated from black spot-diseased fruits of *Phyllanthus emblica* which resulted in the development of whitish-gray mycelium on PDA, with straight, hyaline to light brown color conidiophores and cylindrical oval to ellipsoid light brown conidia arranged in chains or scattered on the surface (Sikder et al., 2021). Generally, a phylogenetic tree illustrates the evolutionary pattern of an organism, and our results depict the simplest phylogenetic tree which was constructed using *Thielaviopsis* isolates obtained from different parts of the pineapple plant, such as diseased fruit, decaying leaves, and soil samples collected from pineapple farming fields. This indicated that they have a common old ancestor, and most isolates diverged from recent common ancestors. As a result, the isolates obtained belong to a monophyletic group; therefore, they can infect pineapple fruits as a complex. Furthermore, the pathogen has the potential to survive in plant debris and soil as a quiescent pathogen until it infects the fruits. Meanwhile, it provides reliable information that the pathogen may infect fruits at harvesting through the cut surface because *Thielaviopsis* is known as a wound pathogen. However, the resulting isolates consisted of *Thielaviopsis paradoxa* which was confirmed by the ITS gene, contradicting the findings of, those who elucidated that the most predominant variety causing black rot disease in pineapple is *Thielaviopsis ethacetica* (Borges et al., 2019).

Furthermore, this study revealed that *Curvularia eragrostidis* and *Trichoderma asperellum* are associated pathogens in black rot-diseased MD2 pineapple fruits, and some previous studies elucidated that *Penicillium* spp. and *Aspergillus niger* have been associated with black rot disease in pineapple fruits (Thelma and Festus, 2018). Moreover, the “crying for help strategy” (Liu and Brettell, 2019) states that plants can perform as an ecological unit comprising the host and all symbionts to enhance their immunity and attract beneficial microbes from the environment to cope with abiotic and biotic stresses confirmed by this study with isolation of *Trichoderma asperellum* from diseased MD2 pineapple fruits, which is well established antagonistic beneficial fungi against *T. paradoxa* (Wijesinghe et al., 2010).

Water loss from fresh produce is directly proportionate to the difference in water vapor pressure between a commodity and the environment (Kader, 2013). This study confirmed that storage at room temperature (30 ± 2°C) and 75% RH resulted in higher disease severity in *T. paradoxa* inoculated MD2 fruits than cold storage (9 ± 2°C and 85% RH). Our results can be confirmed and corroborated by several past research studies which supportively addressed how fresh produce behaves with their storing microenvironment and the impact of spreading postharvest disease after infection. In addition, Tzeng et al. (2010) cited that 25–30°C is the optimum temperature level for hyphae growth of *T. paradoxa*, and they elucidated no growth at low temperatures such as 10°C or higher temperatures of more than 40°C. Similarly, black spot disease caused by *T. paradoxa* in amla fruits (*Phyllanthus emblica*) has shown maximum hyphal growth at 15–35°C (Sikder et al., 2021). *T. paradoxa* isolated from sugarcane sett rot diseased stem cuttings showed scanty growth at 10°C and no growth at 40°C (Majumdar and Mandal, 2018). Furthermore, they reported that moisture in the surrounding environment favors the spores, germination, and penetration of *T. paradoxa* in the host.

Kader (2002) suggested that *T. paradoxa* is unable to sporulate under extreme temperatures, with maximum sporulation occurring at 30°C^,^ followed by 25 and 20°C. Exceptionally, exposing the inoculated fruits to room temperature conditions in the present study could have resulted in water loss and ultimately affected fruit firmness due to shrinkage which may trigger the ability of *T. paradoxa* to penetrate. It has been proven that high temperature causes a high rate of transpiration and may significantly affect the fruit firmness of pineapple, while a low temperature of 9°C does not affect fruit firmness during the storage period (Quyen et al., 2013). Furthermore, harvested fresh pineapple fruits respire faster than fruits in the pre-harvesting stage and ultimately result in metabolic changes which may adversely affect fruit firmness and favor the penetration of fungal hyphae (Chowdhury et al., 2019). However, storage at high temperatures and relative humidity adversely affects the shelf life of fruits, as evidenced by the disease triangle (Kuruppu et al., 2023).

The different inoculum concentrations experimented with in this study revealed that the inoculum concentrations of 2.5 × 10^3^ spores/mL and 5 × 10^3^ spores/mL have potencies to infect healthy fruits under room temperature conditions but are unable to do so under cold storage conditions. The minimum concentration of 1 × 10^3^ spores/mL did not cause any black rot symptoms under either incubation condition. Our findings are in contradiction to past research, which elaborated that no infection by inoculating 10 or 1 × 10^2^ spores/mL and 1 × 10^5^ spores/mL was considered the minimum threshold concentration of inoculum to infect healthy pineapple fruits at room temperature (27°C) (Collins, 1968).

Furthermore, the interaction effect of the five inoculum concentrations and fruit stored under the two micro-environmental conditions was highly significant in terms of mean lesion length and percentage of the mean infected area of inoculated fruits. The findings are in line with those who mentioned that pineapple disease of sugarcane, commonly known as sett rot disease, caused by *T. paradoxa*, shows maximum colony growth at 35°C and maximum sporulation intensity (60.67 × 10^6^ spores/mL) at 28°C and the opposite was observed at 5–20°C (Yadahalli et al., 2007).

The determination of the most susceptible stage of fruits in two harvesting indices focusing on distant marketing (index 0) and local market (index 2) on infection of black rot pathogen revealed that index 2 is the most susceptible stage than index 0 fruits; meanwhile, these results can be supportively discussed by past research studies. All visualized symptoms, such as lesion length, percentage of the infected area, and the calculated DSI (%), showed significantly higher values in index 2 fruits than in index 0 fruits, while pH increased with ripening. The pH of index 0 (T1) and 2 (T2) were 4.24 and 4.36, respectively. These results are supported by the findings of Sikder et al. (2021), who elaborated that the growth of *T. paradoxa* was suppressed at pH 5, intermediate growth was at the pH range 6–8 and the maximum growth was at pH 9. Another study revealed that the maximum growth of *Ceratocystis fimbriata* was at pH 7.5, and mycelial growth declined from pH 5.5 to 2 (Sonyal et al., 2015). The colony growth was gradually increased in pH 3 to 7, and low colony diameter was observed in acidic conditions. Meanwhile, the maximum sporulation was shown at pH 7 and less sporulation was reported under acidic conditions. Hence, it can be used to supportively discuss our results that zero-indexed fruits show less disease severity than index 2 fruits. The changes in physicochemical parameters with ripening, such as sugar composition, antioxidant content, and some amino acids, may enhance the pathogenicity of inoculated fruits (Ding and Syazwani, 2016).

The findings on disease severity on fruits with two different harvesting indices can be corroborated by those who mentioned that ripened pineapple fruits are more susceptible to black rot disease than green fruits due to fulfilling the nutritional requirement for fungal growth (Alao, 2000). They elucidated that after infection by *T. paradoxa* in pineapple fruits, sucrose breaks into glucose and fructose during pathogenesis and is consumed by the pathogen for its growth and sporulation. Subsequently, the pathogen is responsible for altering amino-N metabolism and eliminating all amino acids, except histidine, which is the major organic nitrogen source of the pathogen (Alao, 2000).

Fruit ripening affects the antioxidant content of fruit (Fawole and Opara, 2013). In this study, index 2 fruits with 50% yellow shells and flattened eyes were ripened compared to index 0 fruits with greenish shells and flattened eyes. Consequently, organic acid content and ascorbic acid content may be reduced with the ripening of fruits (Ding and Syazwani, 2016). In contrast, the organic acid content in pineapple is mainly comprised of malic acid and citric acid (Hassan et al., 2011). Ascorbic acid, malic acid, and citric acid are considered antioxidants in pineapple and have a key role in scavenging reactive oxygen species (ROS) and preventing oxidative stress (Lester et al., 2008). It could be suggested that the colonization by the pathogen in index 2 fruits is higher than that in index 0 fruits, which may be due to the reduction of antioxidant properties with ripening. The antioxidant activity provides self-defense to the host from invading pathogens. The Pearson product correlation of the data revealed that moderate and strong positive and statistically significant interaction between the host and external micro-environmental factors lead to the progression of black rot (Yadahalli et al., 2007).

The survival ability of black rot pathogen with decayed leaves revealed that several colonies of *Thielaviopsis paradoxa*, *Ceratocystis paradoxa*, *Thielaviopsis ethecetica, Fusarium* species, and *Lasiodiplodea theobromae* isolates were prominent. Our observations are consistently supported by those who mentioned that butt rot which is a serious pineapple disease caused by *T. paradoxa*, is associated with crowns and contributes to contaminating soil when using infected crowns as a planting material (Rohrbach and Johnson, 2009). It was reported that *T. paradoxa* is a widely distributed soil pathogen that can communicate with plant aerial parts by splashing water by rain, soil particles, or manual operations (Al-Onazi et al., 2011). In addition, it has been documented that pineapple is a year-round producing crop, and ratoon crops are maintained in different stages and promote high incidence and performance of pests and diseases (de Matos, 2017).

Future research should focus on developing advanced diagnostic tools, such as PCR and LAMP, for early detection of black rot pathogens and leveraging genomic and transcriptomic studies to uncover molecular mechanisms of infection and resistance. Studies examining the effects of changing climatic conditions on black rot epidemiology are critical. Understanding how temperature, humidity, and other environmental factors influence pathogen virulence and spread will be vital for predicting outbreaks and devising adaptive management strategies. Exploring host-pathogen interactions and identifying beneficial microbes for biocontrol could pave the way for disease-resistant varieties and eco-friendly management strategies. By addressing these research gaps, future studies can contribute to a comprehensive and sustainable solution to black rot in MD2 pineapples, benefiting both growers and the global pineapple industry.

## 5 Conclusion

Pineapple cultivation plays a vital role in Malaysia’s agricultural sector, requiring robust monitoring to improve production and capitalize on export opportunities. One of the significant challenges faced by growers is black rot disease, primarily caused by *Thielaviopsis paradoxa*, leads to severe postharvest losses. Additional fungal species, such as *C. eragrostidis* and *T. asperellum*, also contribute to the problem. Research shows that even a minimal concentration of fungal inoculum can infect healthy fruits, whether stored at room temperature or in cold storage. Disease severity is influenced by micro-environmental conditions and host-specific factors. The pathogen’s ability to survive in decaying plant material and soil as a dormant agent increases the risk of cross-contamination, posing a challenge to pineapple quality, especially for export markets. To address these issues, it is essential to develop innovative strategies tailored to the specific risks posed by *T. paradoxa*. Such measures would help mitigate the pathogen’s spread, reduce economic losses, and enhance the sustainability of pineapple farming in Malaysia.

## Data Availability

The datasets presented in this study can be found in online repositories. The names of the repository/repositories and accession number(s) can be found within Datasheet 1.

## Footnotes

^1^ https://www.phylo.org/
