# Supplementary material for: Comprehensive analysis of causal pathogens and determinants influencing black rot disease development in MD2 pineapples
Source: Front Microbiol. 2025 Jan 21;15:1514235. doi: 10.3389/fmicb.2024.1514235 (PMC11791799; doi:10.3389/fmicb.2024.1514235)
Supplement: Supplementary file 1 [file Data_Sheet_1.docx]

| **Isolate** | **Fungi** | **Accession numbers** |
| --- | --- | --- |
| JA4 | *Thielaviopsis paradoxa* | MW082788 |
| JA1 | *Aspergillus aculeatus* | MW082785 |
| JA2 | *Trichoderma asperellum* | MW082791 |
| JA3 | *Curvularia eragrostidis* | MW082787 |
| MA1 | *Aspergillus assiutensis* | MW082792 |
| MA2 | *Trichoderma erinaceum* | MW082793 |
| MA3 | *Curvularia eragrostidis* | MW082794 |
| MP1 | *Lasiodiplodea theobromae* | MW082796 |
| MP2 | *Thielaviopsis paradoxa* | MW082790 |
| SA3 | *Aspergillus assiutensis* | MW082805 |
| SA4 | *Trichoderma strigosellum* | MW082808 |
| SP1 | *Neoscytalidium hyalinum* | MW082809 |
| SP2 | *Neoscytalidium dimidiatum* | MW082810 |
| JDL1 | *Lasiodiplodea theobromae* | MZ496555 |
| JDL3 | *Lasiodiplodea theobromae* | MZ496627 |
| JDL5 | *Thielaviopsis paradoxa* | MZ496629 |
| JDL7 | *Thielaviopsis ethacetica* | MZ496633 |
| JDL2 | *Thielaviopsis paradoxa* | MZ496626 |
| J9 | *Fusarium* spp. | MZ505640 |

The list of accession numbers for the datasets within the NCBI.
